# Supplementary material for: Are There Thresholds in Glioblastoma Cell Death Responses Triggered by Temozolomide?
Source: Int J Mol Sci. 2019 Mar 28;20(7):1562. doi: 10.3390/ijms20071562 (PMC6480213; doi:10.3390/ijms20071562)
Supplement: Supplementary file 1 [file ijms-20-01562-s001.pdf]

Supplemental Material

Are there thresholds in glioblastoma cell death responses triggered by temozolomide?

Yang He and Bernd Kaina

Institute of Toxicology, University Medical Center Mainz, Obere ZahlbacherStr. 67, D-55131 Mainz, Germany

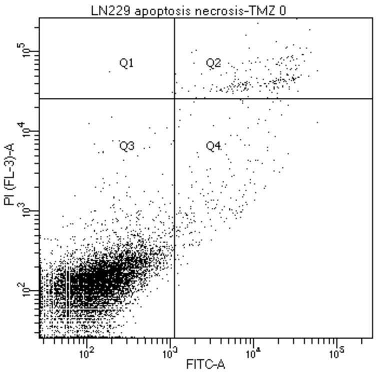

LN-229 control

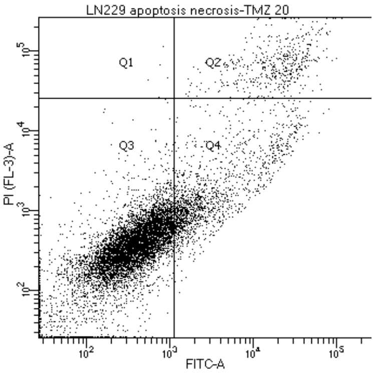

LN-229 TMZ 20 µM

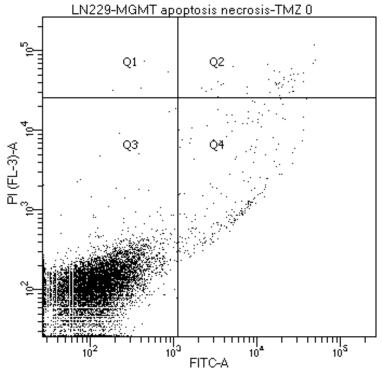

LN-229MGMT control

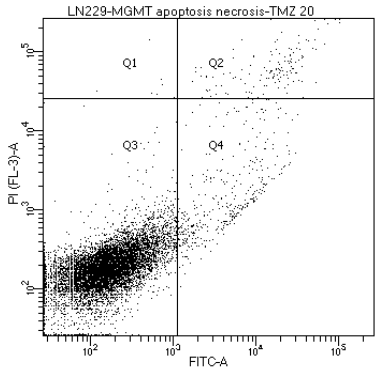

LN-229MGMT TMZ 20 µM

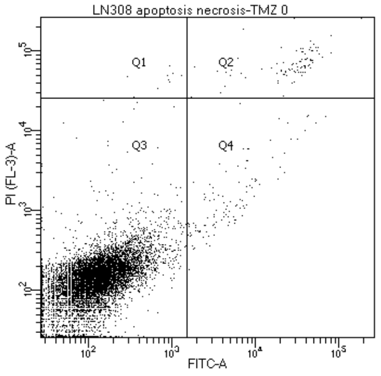

LN-308 control

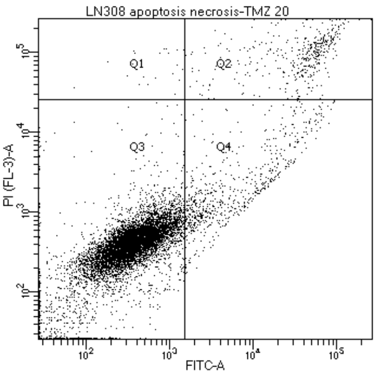

LN-308 TMZ 20 µM

Supplement Fig. S1
Apoptosis and necrosis measured by annexin V/propidium iodide (AV/PI) staining and flow cytometry. Cells were not treated (control) and treated with temozolomide.

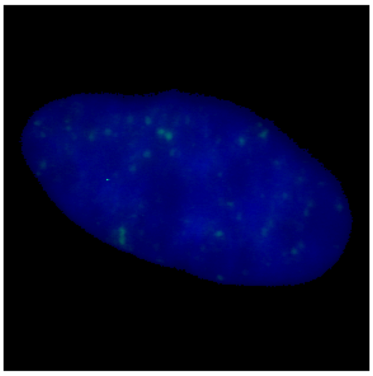

LN-229 control

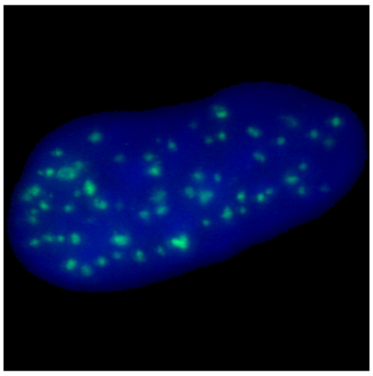

LN-229 TMZ 20 µM

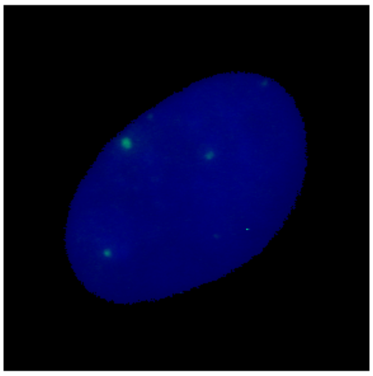

LN-229MGMT control

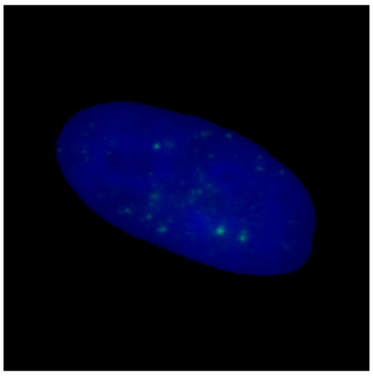

LN-229MGMT TMZ 20 µM

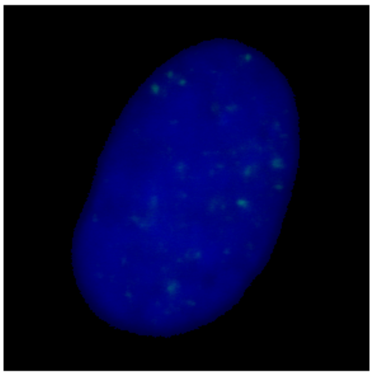

LN-308 control

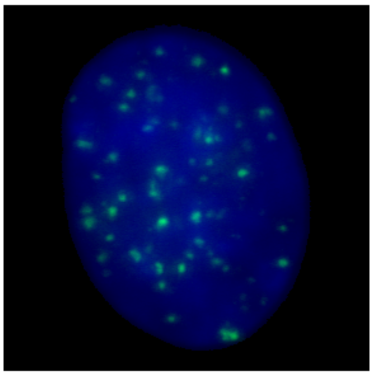

LN-308 TMZ 20 µM

Supplement Fig. S2
γH2AX foci in LN-229, LN-229MGMT and LN-308 cells not treated (control) and treated with temozolomide.

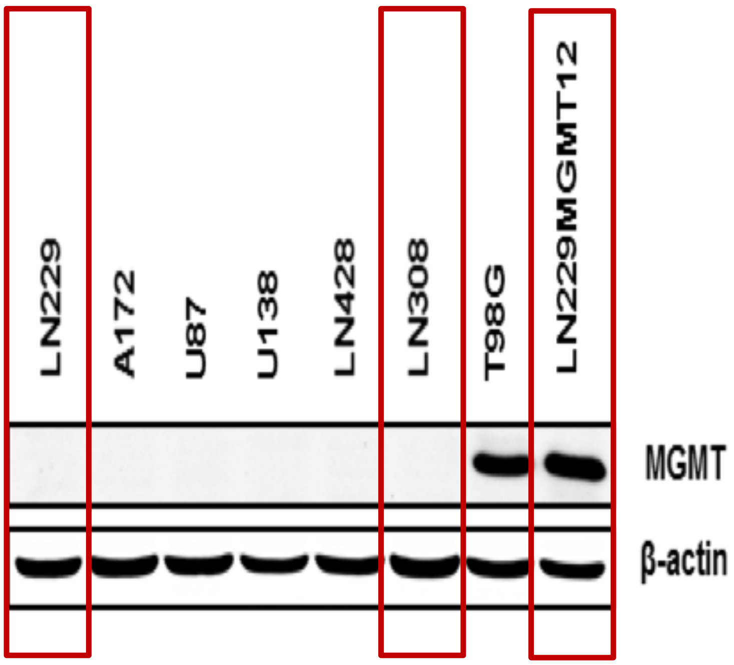

Supplement Fig. S3
MGMT expression in different glioma cell lines determined by Western blot analysis.

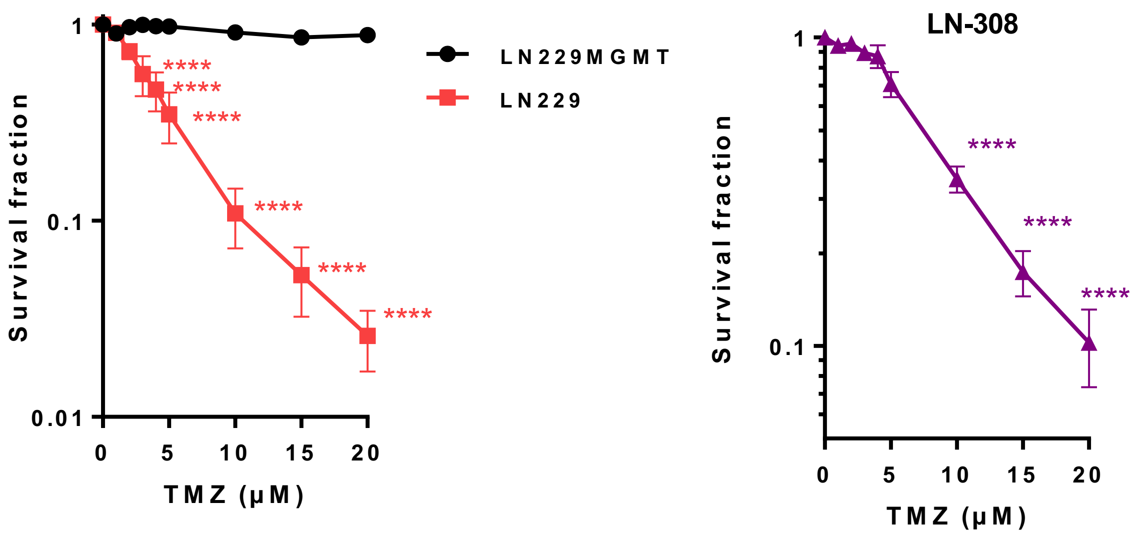

Supplement Fig. S4
Survival (relative colony formation) of LN-229, LN-229MGMT and LN-308 glioblastoma cells treated with temozolomide.
